# Real-Time Plasma Process Condition Sensing and Abnormal Process Detection

**DOI:** 10.3390/s100605703

**Published:** 2010-06-08

**Authors:** Ryan Yang, Rongshun Chen

**Affiliations:** Department of Power Mechanical Engineering, National Tsing Hua University, Hsinchu 30013, Taiwan; E-Mail: ryyanga@yahoo.com.tw

**Keywords:** process/equipment fault detection, spectrum, optic emission spectrum

## Abstract

The plasma process is often used in the fabrication of semiconductor wafers. However, due to the lack of real-time etching control, this may result in some unacceptable process performances and thus leads to significant waste and lower wafer yield. In order to maximize the product wafer yield, a timely and accurately process fault or abnormal detection in a plasma reactor is needed. Optical emission spectroscopy (OES) is one of the most frequently used metrologies in *in-situ* process monitoring. Even though OES has the advantage of non-invasiveness, it is required to provide a huge amount of information. As a result, the data analysis of OES becomes a big challenge. To accomplish real-time detection, this work employed the sigma matching method technique, which is the time series of OES full spectrum intensity. First, the response model of a healthy plasma spectrum was developed. Then, we defined a matching rate as an indictor for comparing the difference between the tested wafers response and the health sigma model. The experimental results showed that this proposal method can detect process faults in real-time, even in plasma etching tools.

## 1. Introduction

### 1.1. Background and Motivations

In the era of nanotechnology in semiconductor manufacturing, the electronic component density is rapidly increasing as device size significantly decreases. Consequently, the manufacturing process flows become more complicated. To achieve device yield improvements, the process windows must be less narrow than the size of the fabricated devices. Also, the design of manufacturing equipment is more complicated due to the smaller process window. As a result, sophisticated semiconductor equipment has to be developed. This equipment should display inherent variability in process condition control because they are composed of individual control components, such as mass-flow controllers, pressure controllers, RF controllers, temperature controllers, and so on. Although a certain amount of inherent variability is unavoidable, in order to maximize the process/wafer yield, any significant process condition shifts must be detected when the variation becomes large, compared to the process background noise. Such shifts are often considered as fault process. When the operating conditions shift beyond an acceptable range, the product yield will be reduced. Thus, timely and accurate fault detections are needed in semiconductor manufacturing [1].

**Figure 1.**
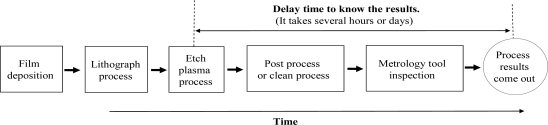
Delay time to know process results in a plasma etch process.

Statistical process control (SPC), a traditional skill, is used to identify out-of-control processes, in which the control charts method is implemented. The acceptable process variation range is defined as the control limit by the statistical method. Any measurement data beyond the control limit is deemed as out-of-control. In this situation, some process diagnosis and corrective actions must be taken to remove the unusual source of variability. Although the SPC method is able to detect the undesired process shifts, it is incapable of detecting the questionable processes and thus cannot stop the on-line process until the off-line/in-line data measurement has occurred. This delay time between the fault process and post process measurements will result in potentially large numbers of wafers/devices that do not meet the required specifications. Figure 1 shows the typical delay time to know the process results in an etch process, at which the silicon wafers are processed and then measured to ensure the process meets the requirements. Often it needs several hours or days between the end of the wafer processing time and the time of data measurement. If the bad process wafers can not be detected in a timely fashion, it will lead to hundreds or even thousands of wafers being scrapped due to the delay time.

This study employed a Transformer Coupled Plasma (TCP) reactor as the test plant. TCP is a successful process kit in the semiconductor field and is a high density and low-pressure plasma process technique. It uses the electromagnetic force to ionize the reactive gases and to induce the chemical process upon the semiconductor wafers, in order to obtain a required pattern or to deposit a thin film. The bias RF system, which can control the plasma ion bombardment force to obtain more vertical profile in etching pattern defined, is applied on the electrostatic chuck (ESC). Since the TCP reactor has high capability of producing tiny features, it is widely used in etching various materials. However, due to a lack of real-time etching control, it often results in some unacceptable process shifting and leads to significant waste and lower yields. For example, Texas Instrument announced that it lost about $135 million annually in its factories because of a lack of effective real-time control and diagnostic equipment in etching processes [2]. Therefore, an excellent etch tool is required for developing a real-time fault detection system.

An *in-situ* process monitoring sensor is a key feature for developing such a kind of system. Optical emission spectroscopy (OES) is one of the most frequently used metrology techniques. An OES system can measure the variation of the optical emission intensity of a plasma, which is affected by the reactants and by-products inside the reacting chamber. This method provides the capability of monitoring the plasma chemistry reactions directly by a non-invasive method. Currently, OES is also applied to etch end-point detection [3]. Even though OES has the advantage of non-invasiveness, it provides a huge amount of information. As a result, the analysis of the data is a big challenge [4,5]. In this study, a digital image processing technique is implemented so that the time series of OES full spectrum intensity is transferred into an image pattern. After collection of the image patterns of healthly plasma conditions, we used a statistical process method to build up a health condition model, called the health sigma model. Comparing the image patterns of the process conditions between the incoming testing data and the normal sigma model, the fault process condition in each recipe step will be found by calculating the matching rate between testing image patterns and the health sigma model. The plasma etching process equipment was implemented for achieving a timely and *in-situ* plasma condition monitor.

### 1.2. Literature Review

Yue *et al.* [6] investigated the suitability of using optical emission spectroscopy (OES) for the fault detection and classification of plasma etchers. The OES sensor system used in [2] can collect spectra at up to 512 different wavelengths. Multiple scans of the spectra are taken from a wafer, and the spectra data are available for multiple wafers. As a result, the amount of the OES data is typically large. This poses a difficulty in extracting relevant information for fault detection and classification. The authors used multiple principal component analysis (PCA) to analyze the sensitivity of the multiple scans within a wafer with respect to typical faults such as etch stop, which is a fault that occurs when the polymer deposition rate is larger than the etch rate. Several PCA-based schemes are tested for the purpose of fault detection and wavelength selection. To construct the final monitoring model, the OES data of selected wavelengths are properly scaled to calculate fault detection indices. However, this work utilized the PCA method to reduce data handling amount and to detect the limit wavelengths. As a result, it sacrificed the detection ability in some events.

In Hong *et al.* [7], neural networks (NNs) have been applied for the fusion of data generated by two *in-situ* sensors: optical emission spectroscopy (OES) and residual gas analysis (RGA). While etching is performed, OES and RGA data are simultaneously collected in real time. Several pre-determined, statistically significant wavelengths (for OES data) and atomic masses (for RGA signals) are monitored. These data are subsequently used for training NN-based time series models of process behavior. Such models, referred to herein as time series NNs (TSNNs), are realized using multilayered perception NNs. Results indicate that the TSNNs not only predict process parameters of interest, but also efficiently perform as sensor fusion of the *in-situ* sensor data. This presented work uses seven specified OES wavelengths and RGA data as its NNs inputs. Consequently, the detection ability of events has been reduced due to the limited number of wavelengths.

Multivariate Statistical Process Control tools have been developed for monitoring and fault detection on a Lam 9600 Metal Etcher in the work of Gallagher *et al.* [8]. Application of these methods is complicated because the process data exhibits large amounts of normal variation that is continuous on some time scales and discontinuous on others. Variations due to faults can be minor in comparison. Several models based on Principal Components Analysis and variants which incorporate methods for model updating have been tested for long term robustness and sensitivity to known faults. Model performance was assessed with about six month’s worth of process data and a set of benchmark fault detection problems. This work used machine variables as their system input parameters. After any component is changed or the equipment is cleaning, this model needs to be built up again for securing the detection ability. Thus, it is hard to implement as a long-term process monitor.

May *et al.* [9] provided a general methodology for the automated diagnosis of integrated circuit fabrication equipment. The technique combines the best aspects of quantitative algorithmic diagnosis and qualitative knowledge-based approaches. Evidence from equipment maintenance history, real-time tool data, and incline measurements are integrated using evidential reasoning. This methodology is applied to the identification of faults in the Lam Research Autoetch 490 automated plasma etching system. Although the model is capable of operating without the metrology data, the quality of the prediction should be degraded.

Gardner *et al.* [10] proposed a new methodology for equipment fault detection. The key features of this methodology are that it allows for the incorporation of spatial information and that it can be used to detect and diagnose equipment faults simultaneously. This methodology consists of constructing a virtual wafer surface from spatial data and using physically based spatial signature metrics to compare the virtual wafer surface to an established baseline process surface in order to detect equipment faults. Statistical distributional studies of the spatial signature metrics provide the justification of determining the significance of the spatial signature. Data collected from a rapid thermal chemical vapor deposition (RTCVD) process and from a plasma enhanced chemical vapor deposition (PECVD) process are used to illustrate the procedures. This method detected equipment faults for all 11 wafers that were subjected to induced equipment faults in the RTCVD process, and even diagnosed the type of equipment fault for 10 of these wafers. However, the proposed method cannot detect the fault in real-time because it uses the metrology measurement data as their analysis input data, which was unable to be generated real-time when the equipment is processing.

To summarize, in [6,7] the authors tried to reduce the OES data amount by using mathematical methods, but this sacrificed part of the event detection ability. The authors of [9,10] used non-*in-situ* metrology tool data as their sensing parameters, therefore their work did not have real-time detection ability. In [8] the system model needs to be rebuilt or it leads to lower fault detection capability and higher false detection possibility if some components are changed. Consequently, it is hard to implement for long-term monitoring. The objective of this work is to develop a system which has following capabilities:

1. Real-time equipment fault detection ability with *in-situ* sensors and a non-invasive method.
2. A simple algorithm which can handle a full spectrum of OES sensor data analysis.
3. High detection capability of different kind of fault events.

## 2. Experimental Apparatus

### 2.1. Transformer Coupled Plasma (TCP) Reactor Etching

**Figure 2.**
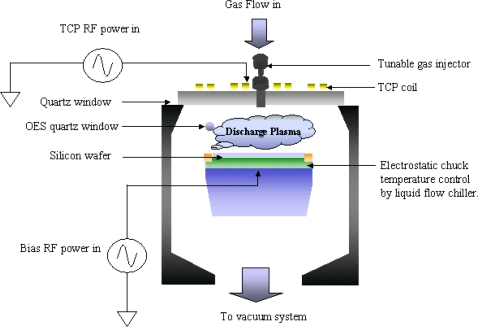
The configuration of the TCP.

A Transformer Coupled Plasma (TCP) reactor, which belongs to high-density-plasma sources shown in Figure 2, can be applied to the etching processes. It uses RF power to activate the TCP coil and thus generate an electromagnetic field to ionize gases and it uses bias RF power to control the ion bombardment force in the wafer chuck to obtain the required etch properties.

A TCP reactor is typically operated below 100 m torr with top and bottom 13.56 MHz RF generators in the TCP coil and electrostatic chuck, respectively. In order to prevent the photoresist pattern from deforming, a backside helium cooling system is used to control wafer temperature during the process stage. The helium gas is the intermediate material to transfer heat to the electrostatic chuck (ESC) and the dielectric liquid, which flows into the ESC, can maintain temperature by using the temperature control unit.

**Figure 3.**
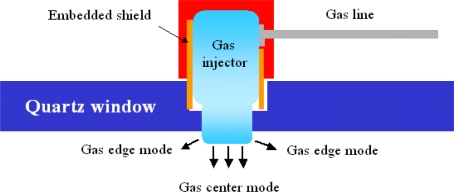
The schematic of three gas injector modes.

The mass flow controllers (MFC) are used to control the flow rate of the process gases in which each type of gas is controlled by an individual MFC. In this study, we used chlorine (Cl_2_), hydrogen bromide (HBr), tetrafluoromethane (CF_4_), oxygen (O_2_), and nitrogen (N_2_) as the process gases. For obtaining better etching properties, a tunable gas injector was also used to control the plasma density. Three different kinds of modes, edge, center, and equal modes, were employed to control the flow distribution, as shown in Figure 3. In edge mode, the most process gas was delivered into the reactor from the edge side of the injector; hence the plasma density increases on the edge side of the wafer. In center mode, the most process gas was injected into the chamber from the center of the injector, resulting in the higher plasma density in the center of the wafer. Finally, in equal mode, the process gas was injected into the chamber from center and edge of the injector, resulting in more uniform plasma density between center and edge of the wafer.

Both chemical and physical mechanisms exist inside the TCP etching reactor. The chemical mechanism is the reactions between the ion gas and the material on the surface of the wafer, including the parts of reactor. The ionized gas is generated by the TCP electromagnetic field and dominates the chemical reaction. On the other hand, the physical ion bombardment of ionized gas increases the speed of chemical reactions by breaking the chemical bonds of the materials on the surface. The RF power applied to the bottom electrodes induces the physical etching process in the etch chamber. Since electrons have higher mobility than ions due to their lesser mass, they are accelerated toward ground and induce the negative DC self-bias voltage between plasma and bottom electrodes. Both reaction mechanisms enable the etch process with a higher etch rate than possible with either independent reaction.

### 2.2. Optical Emission Spectroscopy (OES)

**Figure 4.**
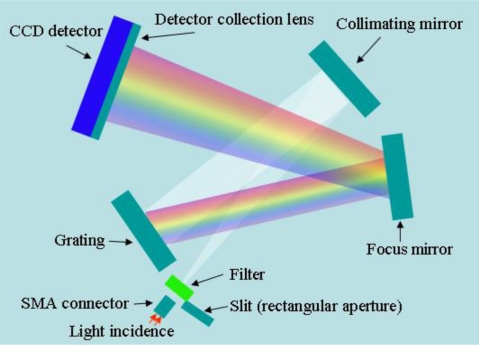
OES module configuration.

OES is an *in-situ* sensor for plasma process monitoring, which does not interfere with the plasma. The plasma emission lights have rich information about the plasma species, which can be used to monitor the etching rate, uniformity, selectivity, critical dimensions, and even the profile of etching features on a wafer. The OES monitoring wavelength covers roughly from 190 to 870 nm and uses a 2,048-pixel CCD array with an optical resolution about 1.3 nm. The fiber optic bundle is made of seven 100-μm fibers terminated on each end by SMA 905 connectors, as shown in Figure 4. Multiple fibers insure that adequate light will continue to be transmitted into the OES unit even if some of the fibers are broken due to excessive stress. The fibers cannot transmit the wavelength of light below 200 nm. Each optical adapter consists of a fiber-packed co-monitoring device with a UV collimating mirror and a connection port for the TCP reactor fiber assembly. If the OES window gets too dirty, light cannot be adequately transmitted into the fibers. In addition, the intensity of CCD reading is affected by the snapping time, set to be 100 ms in this study.

### 2.3. Data Collection

**Figure 5.**
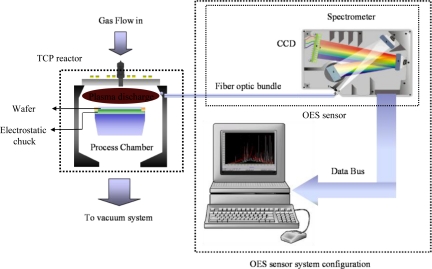
Overall system configurations.

Figure 5 illustrates the overall system configuration, consisting of a TCP reactor, OES module, and computer. The plasma emission beam is sent out from the OES window and is incident upon the OES module through the optic fiber.

**Figure 6.**
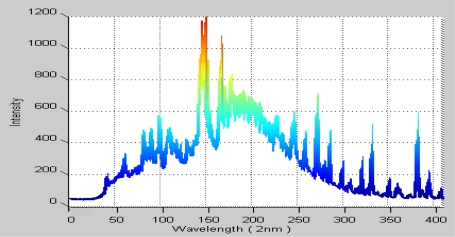
Spectrum data chart in one sample period.

The grating spectrum device (Figure 5) splits out the plasma light arranged in order with its wavelength into the CCD unit. Next, the CCD senses the wavelength intensity and transfers the data, which can be represented as in Figure 6, to the computer for further analysis.

**Figure 7.**
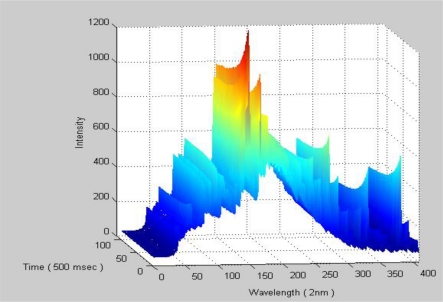
Spectrum data integrated with time.

After collecting full recipe step data, the spectrum data is integrated time by time. Figure 7 is an example of the represented data where the full process response, illustrated by plasma spectrum, consists of transient and steady states. In the transient state, the molecular/atom is excited and ionized by RF power, in which the plasma condition are not stable and may induce plasma spiking, RF reflected power, pressure unstable, and so on. In the nano-scale device manufacture era, this kind of state must be considered due to the less critical process window. However, it is not easy to handle such an amount of process data by a simple computation method. For controlling the data amount, the sampling time of this system is 500 ms.

### 2.4 Design of Experiments (DOE)

**Figure 8.**
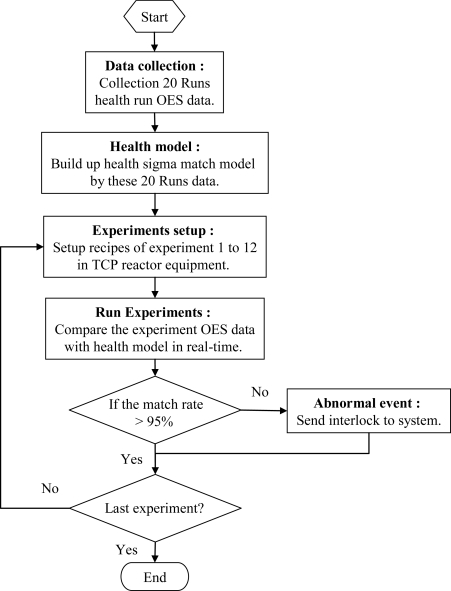
Flowchart of the experiment.

The bare silicon wafer, which is used as a common wafer in polysilicon gate etching, is used as the etched material. Also, to reduce the memory effect of process conditions, the waferless auto clean (WAC) recipe is applied to every wafer before every experiment for removing the polymer deposited on the chamber wall during processing. Figure 8 shows the flowchart of the experiment. At the beginning, it needs collect 20 runs of healthy condition OES data and then uses these data to build up a health model. Then, we compare the data of these experiments with the health model and calculate the match rate of a test wafer. If the match rate is less than 95%, it means there was something wrong with this test wafer when it was processed. Then the system will send out a signal to stop the tool to prevent more wafers from running with bad process conditions.

The process recipe parameters consist of Cl_2_, HBr, CF_4_, O_2_, N_2_, TCP RF power, bias RF power, pressure, temperature, gas injection ratio mode, and so on. For testing the sensitivity of each process physical components, DOE can be cataloged as gas flow shift, RF power variance, chamber pressure shift, temperature shift, gas injected ratio changed and chamber leakage detection. Therefore we designed 12 experiments for the event detection above. In each experiment some of the parameters will be changed. Table 1 shows the experiment sequence for Experiments 1 through 12 with the changing parameters in each experiment. In additions, the standard recipe is inserted between two experiments since we need confirm the fault detection which is not caused by the chamber condition shifted.

In gas flow shift-testing in Experiments 1 to 5; the Cl_2_, HB_r_, CF_4_, Cl_2_ and HBr, and Cl_2_ and CF_4_ gases are increased by 0.5%, 1%, 2%, 3%, 4% and 5% ratio of standard-run setting flow rate, respectively. In RF power changed tests, the TCP and bias RF power also increased by 0.5%, 1%, 2%, 3%, 4% and 5% ratio of standard-run setting RF power in Experiments 6 and 7, respectively. For the pressure shift test experiment, the chamber pressure is increased by 2%, 5%, 10%, 15%, 20% and 25% ratio of standard-run setting in Experiment 8. There are six pieces of silicon wafers used in each of the above experiments. Besides, the gas injection ratio tests are shown in Experiments 9 and 10 for testing the indicator ability of plasma concentration distribution shift condition. Finally, the chamber leakage from atmosphere event (add N_2_ in process) and ESC temperature shift event are designed in Experiment 11 and 12, respectively. All of above experiments are the usually machine failed cases in etch tools. Boldface parts in Table 1 show the differences from the standard-run recipe.

## 3. Fault Detection Method of Plasma Process Condition

Some environment light enters the chamber from the quartz window or view port quartz, which will induce some background noise signal in the OES system. The plasma conditions have inherent process condition control variability because they are composed of some individual control components, such as mass-flow controllers, pressure controller, RF controllers, temperature controllers, and so on. These variances also generate the OES background noise signal.

To stabilize the fault process detection capability, we propose a health plasma behaviors modeling method, which is called sigma model of spectrum response in this study. Equations 1 to 4 describe these OES data, illustrating as a time series matrix from t = 0 to n seconds:

(1) $$S_{0}={\;\text{Plasma OES Data}}_{t=0}={\left[r_{01}\;r_{02}\ldots \;r_{02048}\right]|}_{t=0}$$

(2) $$S_{1}={\;\text{Plasma OES Data}}_{t=\;0.5\;\text{sec}}={\left[r_{11}\;r_{12}\ldots \;r_{12048}\right]|}_{t=0.5\;\text{sec}}$$

(3) $$\begin{array}{c} S_{2}={\;\text{Plasma OES Data}}_{t=1\;\text{sec}}={\left[r_{21}\;r_{22}\ldots \;r_{22048}\right]|}_{t=1\text{sec}}\\ \vdots \end{array}$$

(4) $$S_{2n}={\;\text{Plasma OES Data}}_{t=n\text{sec}}={\left[r_{2n1}\;r_{2n2}\ldots \;r_{2n2048}\right]|}_{t=n\text{sec}}$$

where *r*_2*n*1_, *r*_2*n*2_, … and *r*_2*n*2048_ are the intensities of specified wavelengths of plasma emission light at t = n seconds, *S_2n_* is the full spectrum response at t = n seconds. In this study, the sampling rate of OES data is 500 ms. The plasma process is on going with recipe setting time by time. After collecting all the OES data of a run, we can write the full step OES response data as Equations 5 to 7, where *R*_1_, *R*_2_, and *R*_m_, are the first, second, and mth health plasma response run, respectively:

(5) $$\begin{array}{c} R_{1}=\mathrm{The}\;\mathrm{OES}\;\mathrm{Response}\;\mathrm{Data}\;\mathrm{of}\;\mathrm{Run}\;1\;=\;{\left[\begin{array}{c} S_{0R}\\ S_{1R}\\ \vdots \\ S_{(2n-1)R}\\ S_{2\mathrm{nR}} \end{array}\right]}_{\mathrm{run}1}\\ ={\left[\begin{array}{ccccc} r_{01R} & r_{02R} & \cdots & r_{0(L-1)R} & r_{0\mathrm{LR}}\\ r_{11R} & r_{12R} & \cdots & r_{1(L-1)R} & r_{1LR}\\ \vdots & \vdots & \ddots & \vdots & \vdots \\ r_{(2n-1)1R} & r_{(2n-1)2R} & \cdots & r_{(2n-1)(L-1)R} & r_{(2n-1)\mathrm{LR}}\\ r_{2n1R} & r_{2n2R} & \cdots & r_{2n(L-1)R} & r_{2\mathrm{nLR}} \end{array}\right]}_{L=2048,R=1} \end{array}$$

(6) $$\begin{array}{c} R_{2}=\mathrm{The}\;\mathrm{OES}\;\mathrm{Response}\;\mathrm{Data}\;\mathrm{of}\;\mathrm{Run}\;2\;=\;{\left[\begin{array}{c} S_{0R}\\ S_{1R}\\ \vdots \\ S_{(2n-1)R}\\ S_{2\mathrm{nR}} \end{array}\right]}_{\mathrm{run}2}\\ \begin{array}{l} ={\left[\begin{array}{ccccc} r_{01R} & r_{02R} & \cdots & r_{0(L-1)R} & r_{0\mathrm{LR}}\\ r_{11R} & r_{12R} & \cdots & r_{1(L-1)R} & r_{1\mathrm{LR}}\\ \vdots & \vdots & \ddots & \vdots & \vdots \\ r_{(2n-1)1R} & r_{(2n-1)2R} & \cdots & r_{(2n-1)(L-1)R} & r_{(2n-1)\mathrm{LR}}\\ r_{2n1R} & r_{2n2R} & \cdots & r_{2n(L-1)R} & r_{2\mathrm{nLR}} \end{array}\right]}_{L=2048,R=2}\\ \vdots \end{array} \end{array}$$

(7) $$\begin{array}{c} R_{m}=\mathrm{The}\;\mathrm{OES}\;\mathrm{Response}\;\mathrm{Data}\;\mathrm{of}\;\mathrm{Run}\;m=\;{\left[\begin{array}{c} S_{0R}\\ S_{1R}\\ \vdots \\ S_{(2n-1)R}\\ S_{2\mathrm{nR}} \end{array}\right]}_{\mathrm{runm}}\\ ={\left[\begin{array}{ccccc} r_{01R} & r_{02R} & \cdots & r_{0(L-1)R} & r_{0\mathrm{LR}}\\ r_{11R} & r_{12R} & \cdots & r_{1(L-1)R} & r_{1\mathrm{LR}}\\ \vdots & \vdots & \ddots & \vdots & \vdots \\ r_{(2n-1)1R} & r_{(2n-1)2R} & \cdots & r_{(2n-1)(L-1)R} & r_{(2n-1)\mathrm{LR}}\\ r_{2n1R} & r_{2n2R} & \cdots & r_{2n(L-1)R} & r_{2\mathrm{nLR}} \end{array}\right]}_{L=2048,R=m} \end{array}\;$$

After collecting m runs process data, we can build the plasma health model as Equation 8:

(8) $${\sigma M}_{h}=\bar{R}\pm {3R}_{\sigma }$$

where *σM_h_* represent the health sigma model of OES data, *R̄* is the mean values matrix of *R*_1_, *R*_2_, … to *R_m_*. *R_σ_* is the standard deviation matrix of *R*_1_, *R*_2_, … to *R_m_*. Equations 9 and 10 show the detailed operations. In this study we collect 20 health runs data, or *M* = 20, to build up the health sigma model:

(9) $$\begin{array}{ll} \bar{R} & =\mathrm{The}\;\mathrm{Mean}\;\mathrm{Value}\;\mathrm{of}\;\mathrm{These}\;\mathrm{Health}\;\mathrm{Run}=\frac{\sum_{m=1}^{M}R_{m}}{M}\\ & =\frac{1}{M}\sum_{R=1}^{M}{\left[\begin{array}{ccccc} r_{01R} & r_{02R} & \cdots & r_{0(L-1)R} & r_{0\mathrm{LR}}\\ r_{11R} & r_{12R} & \cdots & r_{1(L-1)R} & r_{1\mathrm{LR}}\\ \vdots & \vdots & \ddots & \vdots & \vdots \\ r_{(2n-1)1R} & r_{(2n-1)2R} & \cdots & r_{(2n-1)(L-1)R} & r_{(2n-1)\mathrm{LR}}\\ r_{2n1R} & r_{2n2R} & \cdots & r_{2n(L-1)R} & r_{2\mathrm{nLR}} \end{array}\right]}_{L=2048}\\ & ={\left[\begin{array}{ccccc} {\bar{r}}_{01} & {\bar{r}}_{02} & \cdots & {\bar{r}}_{0(L-1)} & {\bar{r}}_{0L}\\ {\bar{r}}_{11} & {\bar{r}}_{12} & \cdots & {\bar{r}}_{1(L-1)} & {\bar{r}}_{1L}\\ \vdots & \vdots & \ddots & \vdots & \vdots \\ {\bar{r}}_{(2n-1)1} & {\bar{r}}_{(2n-1)2} & \cdots & {\bar{r}}_{\left(2n-1)(L-1\right)} & {\bar{r}}_{(2n-1)L}\\ {\bar{r}}_{2n1} & {\bar{r}}_{2n2} & \cdots & {\bar{r}}_{2n(L-1)} & {\bar{r}}_{2\mathrm{nL}} \end{array}\right]}_{L=2048} \end{array}$$

(10) $$\begin{array}{l} R_{\sigma }=\mathrm{The}\;\mathrm{Standard}\;\mathrm{Deviation}\;\mathrm{of}\;\mathrm{These}\;\mathrm{Health}\;\mathrm{Runs}\;=\;\\ {\left[\begin{array}{cccc} \sqrt{\frac{{\sum_{R=1}^{M}\left({\bar{r}}_{01}-r_{01R}\right)}^{2}}{M}} & \sqrt{\frac{{\sum_{R=1}^{M}\left({\bar{r}}_{02}-r_{02R}\right)}^{2}}{M}} & \cdots & \sqrt{\frac{{\sum_{R=1}^{M}\left({\bar{r}}_{0L}-r_{0\mathrm{LR}}\right)}^{2}}{M}}\\ \sqrt{\frac{{\sum_{R=1}^{M}\left({\bar{r}}_{11}-r_{11R}\right)}^{2}}{M}} & \sqrt{\frac{{\sum_{R=1}^{M}\left({\bar{r}}_{12}-r_{12R}\right)}^{2}}{M}} & \cdots & \sqrt{\frac{{\sum_{R=1}^{M}\left({\bar{r}}_{1L}-r_{1\mathrm{LR}}\right)}^{2}}{M}}\\ \vdots & \vdots & \ddots & \vdots \\ \sqrt{\frac{{\sum_{R=1}^{M}\left({\bar{r}}_{2n1}-r_{2n1R}\right)}^{2}}{M}} & \sqrt{\frac{{\sum_{R=1}^{M}\left({\bar{r}}_{2n2}-r_{2n2R}\right)}^{2}}{M}} & \cdots & \sqrt{\frac{{\sum_{R=1}^{M}\left({\bar{r}}_{2\mathrm{nL}}-r_{2\mathrm{nLR}}\right)}^{2}}{M}} \end{array}\right]}_{L=2048} \end{array}$$

We define the normal plasma condition in any OES response data, of which values deviate within the range *R̄* ± 3*R_σ_*. For stabilizing the fault process detection and minimizing the false alarm probability, we define an indicator, at which we call process match rate as in Equation 11:

(11) $$\mathrm{Match}\;\mathrm{Rate}\;\%=\;\frac{2n\times \;2048\;-\;\#\;\mathrm{of}\;\mathrm{test}\;\mathrm{data}\;\mathrm{which}\;\mathrm{is}\;\mathrm{out}\;\mathrm{of}\;\sigma M}{\mathrm{Size}\;\mathrm{of}\;\mathrm{Process}\;\mathrm{Run}\;\mathrm{Data}\;2n\;\times \;2048}\times 100\%$$

**Figure 9.**
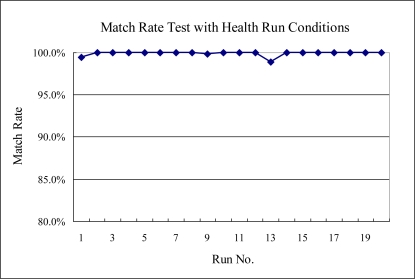
The match rate of the health process data compared to the sigma model.

For testing the match rate of the health data process, we use the original 20 healthy runs data and calculate the match rate as shown in Figure 9. The results demonstrated that the model presented has a very high match rate and very low possibility of false fault detection. A high match rate means more stable processes, high process control ability, and high product quality.

## 4. Results and Discussion

**Figure 10.**
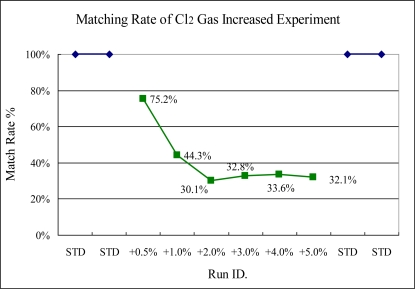
Experiment 1, Cl_2_ variance tested from 50.25 to 52.25 sccm [standard (STD) = 50 sccm].

Experiments 1 to 3 are the shift tests of single gas flow rate. Figure 10 shows that the responding match rate can demonstrate the variance in the plasma conditions significantly, compared to the standard runs.

**Figure 11.**
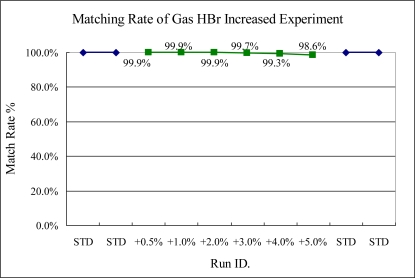
Experiment 2, HBr variance tested from 251.25 to 262.5 sccm (STD = 250 sccm).

The Cl_2_ gas variance test also can be detected by this scheme easily, even the shift only increases 0.5% of standard setting. In this figure, it can be seen that the match rate gets lower when the variance became larger. However, in Experiment 2, the test for HBr variance cannot be checked by this method as shown in Figure 11.

**Figure 12.**
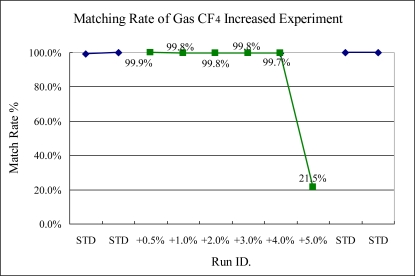
Experiment 3, CF_4_ variance tested from 70.35 to 73.5 sccm. (STD = 70 sccm).

On the other hand, in Figure 12, the CF_4_ gas shift can only be detected when the variance becomes greater than 5%. This proposed method shows a lower capability of detection in the increase of single gas flow rates in the experiments. This is because increasing the single gas flow tests only increases the intensity of limited specific wavelengths, especially in HBr gas increasing experiments, which was unable to be reflect by the proposed method.

**Figure 13.**
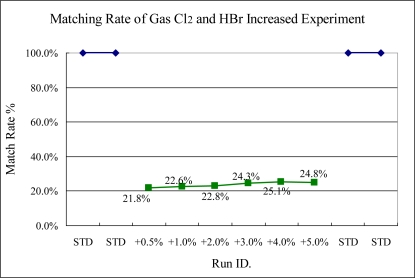
Experiment 4, Cl_2_ and HBr increased at the same time tested (Cl_2_ from 50.25 to 52.25 sccm, STD = 50 sccm; HBr from 251.25 to 262.5 sccm; STD = 250 sccm)

**Figure 14.**
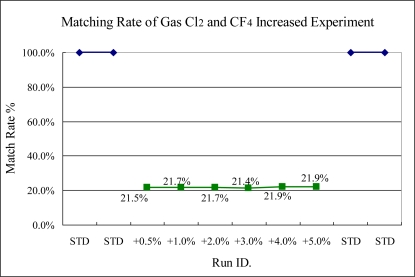
Experiment 5, Cl_2_ and CF_4_ variance at the same time. (Cl_2_ from 50.25 to 52.25 sccm, STD = 50 sccm; CF_4_ from 70.35 to 73.5 sccm; STD = 70 sccm).

The results of Experiments 4 and 5, shown in Figures 13 and 14, respectively, illustrate two kinds of gas flow rates changed at the same time. From the results, the sigma match method can sense these variances significantly because the intensity of specific wavelengths is changed more.

**Figure 15.**
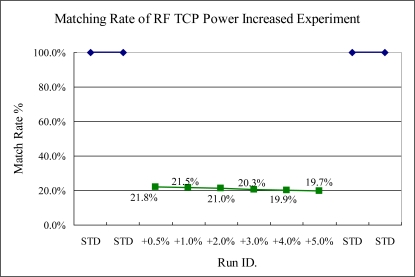
Experiment 6, TCP RF power variance tested from 502.5 to 525 W. (STD = 500 W).

**Figure 16.**
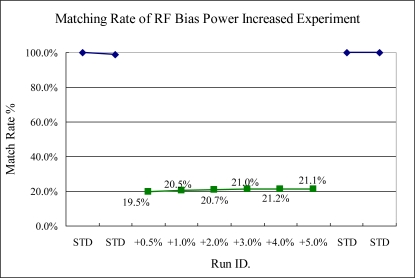
Experiment 7, bias RF power variance tested from 70.35 to 73.35 W. (STD = 70 W).

In RF power sensitivity tests, the TCP and bias RF power variances can be detected. Especially in the TCP RF power change, the sigma match rate indicator trends down, as shown in Figures 15 and 16. This was caused by the ionized gas reaction, increasing by the TCP and bias RF power. In both TCP RF and bias RF, the intensity of spectra is increased significantly, thus the sigma match rate method can detect them, even though only 0.5% RF power is changed.

**Figure 17.**
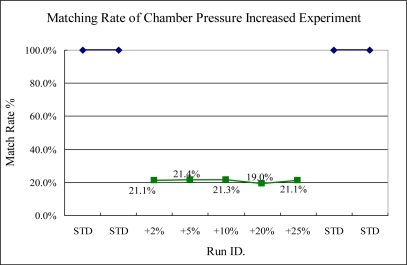
Experiment 8, chamber pressure servo variance tested from 7.14 to 8.75 mtorr. (STD = 7 mtorr).

In the tests of chamber pressure sensitivity, the sigma match rate method is able to show the variance at the beginning of the test, in which the variance of chamber pressure is shift about 2%, compared to the standard-run process, as illustrated in Figure 17. The indicator can detect the chamber pressure shift that is 2% bigger than the standard-run. The increasing pressure implies that the gas density increases. Therefore, the intensity of spectra will be changed.

**Figure 18.**
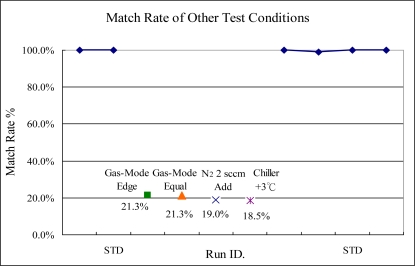
Experiments 9 to 10, gas delivery mode edge changed from equal to edge and center, respectively. Experiment 11, shows adding 2 sccm N_2_ into process chamber and experiment 12 shows adjusting ESC temperature from 60 to 63 degrees.

The test results of the gas injection/delivery ratio change are shown in Figure 18 (standard condition is center mode), where the significant changed in the indicator is observed. Changing gas mode results in gas distribution changes on the edge and center side, which will affect the plasma distribution on these locations.

Since the OES sensor is fixed on top chamber wall and whole spectra are changed, the indicator can detect this event. Also, in chamber leakage test, when we added extra 2 sccm nitrogen into the process chamber, the indicator can display the variance substantially. This is just like a simulation of a chamber leakage event from the atmosphere. At the same time, it causes the extra nitrogen to generate the specific spectra in the OES sensor. Finally, in the experiments of ESC temperature shift, the failed case can be detected by the indicator because the higher ESC temperature will increase the speed of plasma reaction when the temperature of silicon wafer is raised. The detection capability of this proposed method is summarized in Table 2.

## 5. Conclusions

To accomplish real-time and *in-situ* fault process detection, this study employed a plasma condition matrix, comparing with the healthy plasma model by using the time series of OES full spectrum intensity data. An indicator, which is called sigma model matching rate, was employed to show the differences between the normal and abnormal plasma conditions. Twelve experiments have been conducted for detecting the capability of the events and the experimental results showed that the proposed sigma model matching rate method can successfully detect most of abnormal plasma conditions in real-time and prevent low yield and scrap of mass wafers.

## Figures and Tables

**Table 1.** Design of experiments (DOE) for process shift detection.

**Design of experiment (DOE) Exp. description**		**Pressure (mtorr)**	**TCP RF Power (w)**	**Bias RF Power (w)**	**Gas Injection Ratio Mode**	**ESC Temperature (°C)**	**Cl_2_ (sccm)**	**HBr (sccm)**	**CF_4_ (sccm)**	**N_2_ (sccm)**	**Process Wafer #**
Experiment 1	Cl_2_ sensitivity test	7	500	90	Center	60	**50.25∼52.5**	250	70	0	10
Experiment 2	HBr sensitivity test	7	500	90	Center	60	50	**251.25∼262.5**	70	0	10
Experiment 3	CF_4_ sensitivity test	7	500	90	Center	60	50	250	**70.35∼73.5**	0	10
Experiment 4	Cl_2_ and HBr sensitivity test	7	500	90	Center	60	**50.25∼52.5**	**251.25∼262.5**	70	0	10
Experiment 5	Cl_2_ and CF_4_ sensitivity test	7	500	90	Center	60	**50.25∼52.5**	250	**70.35∼73.5**	0	10
Experiment 6	TCP power sensitivity test	7	**502.5∼525**	90	Center	60	50	250	70	0	10
Experiment 7	Bias power sensitivity test	7	500	**90.45∼94.5**	Center	60	50	250	70	0	10
Experiment 8	Pressure sensitivity test	**7.14∼8.75**	500	90	Center	60	50	250	70	0	9
Experiment 9	Gas Mode sensitivity test I	7	500	90	**Edge**	60	50	250	70	0	3
Experiment 10	Gas Mode sensitivity test II	7	500	90	**Equal**	60	50	250	70	0	1
Experiment 11	Add N_2_ 2 sccm	7	500	90	Center	60	50	250	70	**2**	1
Experiment 12	ESC + 3 degree	7	500	90	Center	**63**	50	250	70	0	3
Standard	Standard Recipe	7	500	90	Center	60	50	250	70	0	3

**Table 2.** Summary table for detection capability of events.

**Design of experiment (DOE) Exp. description**		**Event detection ability**		**Event detection sensitivity**		
		**Yes**	**No**	**High**	**Normal**	**Low**
Experiment 1	Cl_2_ sensitivity test	◎			◎	
Experiment 2	HBr sensitivity test		◎	NA	NA	NA
Experiment 3	CF_4_ sensitivity test	◎				◎
Experiment 4	Cl_2_ and HBr sensitivity test	◎		◎		
Experiment 5	Cl_2_ and CF_4_ sensitivity test	◎		◎		
Experiment 6	TCP power sensitivity test	◎		◎		
Experiment 7	Bias power sensitivity test	◎		◎		
Experiment 8	Pressure sensitivity test	◎		◎		
Experiment 9	Gas Mode sensitivity test I	◎		◎		
Experiment 10	Gas Mode sensitivity test II	◎		◎		
Experiment 11	Add N_2_ 2 sccm	◎		◎		
Experiment 12	ESC + 3 degree	◎		◎		
